# Variable Universe Fuzzy–Proportional-Integral-Differential-Based Braking Force Control of Electro-Mechanical Brakes for Mine Underground Electric Trackless Rubber-Tired Vehicles

**DOI:** 10.3390/s24092739

**Published:** 2024-04-25

**Authors:** Jian Li, Yuqiang Jiang

**Affiliations:** School of Mechatronic Engineering, China University of Mining and Technology, Xuzhou 221116, China; lijian0609seu@163.com

**Keywords:** autonomous electric vehicle, brake-by-wire, electro-mechanical braking, clamping force, multi-close-loop VUF-PID controller, cascaded PID, contact point detection

## Abstract

Currently, the main solution for braking systems for underground electric trackless rubber-tired vehicles (UETRVs) is traditional hydraulic braking systems, which have the disadvantages of hydraulic pressure crawling, the risk of oil leakage and a high maintenance cost. An electro-mechanical-braking (EMB) system, as a type of novel brake-by-wire (BBW) system, can eliminate the above shortcomings and play a significant role in enhancing the intelligence level of the braking system in order to meet the motion control requirements of unmanned UETRVs. Among these requirements, the accurate control of clamping force is a key technology in controlling performance and the practical implementation of EMB systems. In order to achieve an adaptive clamping force control performance of an EMB system, an optimized fuzzy proportional-integral-differential (PID) controller is proposed, where the improved fuzzy algorithm is utilized to adaptively adjust the gain parameters of classic PID. In order to compensate for the deficiency of single-close-loop control and adjusting the brake gap automatically, a cascaded three-closed-loop control architecture with force/position switch technology is established, where a contact point detection method utilizing motor rotor angle displacement is proposed via experiments. The results of the simulation and experiments indicate that the clamping force response of the proposed multi-close-loop Variable Universe Fuzzy–PID (VUF-PID) controller is faster than the multi-closed-loop Fuzzy–PID and cascaded three-close-loop PID controllers. In addition, the chattering of braking force can be suppressed by 17%. This EMB system may rapidly and automatically finish the operation of the overall braking process, including gap elimination, clamping force tracking and gap recovery, which can obviously enhance the precision of the longitudinal motion control of UETRVs. It can thus serve as a BBW actuator of mine autonomous driving electric vehicles, especially in the stage of braking control.

## 1. Introduction

In the immediate future, coal resources must continue to account for the majority of energy consumption in China and continue to be widely used all over the world [1]. Underground electric trackless rubber-tired vehicles (UETRVs) are efficient and green auxiliary vehicles in underground coal mine production, completing the task of transporting persons or materials between the surface and underground efficiently and automatically. However, the existing braking systems of mine UETRVs generally use a hydraulic disk braking structure [2]. Although traditional hydraulic braking (THB) systems are currently widely applied, novel brake-by-wire (BBW) systems have obvious advantages such as a simple structure, a faster response under a lower temperature, fewer components and no hydraulic pipes, and a lower potential pollution risk of oil leakages [3]. In addition, the existing electro-hydraulic braking (EHB) applied in the automotive industry is based on electro-boosters, whose power is generated by the engine, meaning that that it is impossible to integrate this type of system into an intelligent chassis control system of novel mine unmanned electric vehicles. Last but not least, high-level automated driving for UETRVs requires a fully decoupled BBW system which can provide more accurate motion control performance during the autonomous braking process. Therefore, it is highly necessary to consider adopting a novel BBW system and a relevant control method in order to deal with the above problems [3,4].

Although there are some advantages related to EMB, it is a pity that it remains at the stage of theory research and bench testing because of unsolved reliability problems when applying EMB in the automotive industry [5]. Meanwhile, there are no mature EMB system products sold on the market currently because of a lack of redundancy for the braking system. Prototype development is only undertaken by the leaders of the automotive industry, such as Bosch and Siemens, because the key technology is very challenging. However, when the initial braking velocity of a vehicle is lower than 50 km/h, the reliability of the EMB system can be acceptable [6,7]. Hence, an EMB consisting of a motor and pure mechanical transmission parts controlled by a high-performance micro controller unit (MCU) is considered to be more appropriate and reliable for use as a braking apparatus for UETRVs and unmanned UETRVs [4].

There have been some previous studies on EHB systems which can provide ideas for solutions for our proposed control method. It has been suggested that PI and a linear quadratic regulator (LQR) could be combined to control the positive pressure of EMB systems in coal mine hoists [8] and achieve an approximately linear relationship between the motor voltage and positive pressure; however, this is only adaptable to vertical lifting equipment in mines. Jin and Feng et al. [9] built an EMB bench and tested both the open-loop control method and the single close-loop force control method on this bench. For the purposes of improving the tracking control ability of the braking force, He Cheng et al. [10] designed a braking torque closed-loop control method based on a switch reluctance motor (SRM), which can achieve fast close-loop regulation. For the purposes of solving the nonlinear and disturbance problems of EMB systems, Soohyeon Kwon et al. [11] considered EMB to be affected by both linear and nonlinear aspects and designed a force estimator based on the Kalman filter (KF). Zhao et al. [12] proposed a slide-mode reaching law to deal with the load torque disturbance of EMB. In order to enhance the adaptive tracking performance of the clamping force of EMB, a single clamping force close-loop controller based on the Variable Universe Fuzzy–PID (VUF-PID) algorithm is proposed to reduce the adjustment time of the clamping force [13], but it always required additional time to adjust the parameters of VUF-PID to track the variable input force command, which is not appropriate under the conditions of anti-block system (ABS) control [14,15]. A kind of automobile power-by-wire steering control method based on FPIDBS control is adopted in Ref. [16], which uses the output of a PID controller as the input of a BS controller, and the parameters of the PID controller are dynamically adjusted based on a fuzzy algorithm. This BS algorithm combined with fuzzy–PID has better performance than an ordinary BS algorithm in resisting other disturbances to the chassis system. In Ref. [17], a new AFSPIDF algorithm based on the fusion of SMC, fuzzy and PID algorithms is proposed to control an active suspension system, and it can realize the nonlinear control theory and suppress the chattering problem of the sliding mode controller through the fuzzy algorithm, which reduces the vibration of the car body.

Many scholars focus on the performance optimization and practical implementation of EHB systems [18], which can provide ideas regarding control methods for the design of this EMB system. In order to deal with the uncertainties and disturbances related to EHB systems, Shi and Huang et al. [19] proposed a dual-loop braking pressure control method with speed and current tracking controllers. In [20], an adaptive sliding-mode controller combining desired-state and integral antiwindup compensation facilitated the improvement of the system’s steady tracking performance. Also, in order to deal with actual friction problems, in [21], linearizing the nonlinear Tustin friction model improved the pressure-tracking accuracy, a sliding mode controller was utilized to suppress friction disturbances, and subsequently, the Lyapunov method was used to verify its stability. Ref. [22] utilized the Lugre friction model to reduce the negative effects of system friction on cylinder pressure tracking accuracy.

It is known that the response speed of an EHB system must be slower than EMB when adopting the same control method because of the inevitable pressure transfer delay time in hydraulic pipelines. Therefore, the EMB system should replace the EHB system in the future [3,18]. Considering the demand of high accuracy of the braking system for coal mine engineering vehicles, both the force loop, current loop and speed loop must be adopted in the overall controller design. Although scholars have proposed a clamping force control method based on intelligent algorithms such as fuzzy or neural networks, these controllers always act on the single-force close-loop unit, which can not produce a balance between the stable state tracking of clamping force and response speed [10,12,13,23]. The current disturbance correction caused by nonlinear load effect can not be suppressed in a timely manner [19]. Additionally, the braking gap should be automatically adjusted by the active braking system, although it is ignored by many researchers. Importantly, it is the first time that the proposed EMB system in this paper has been designed and applied in the UETRV so that basic and reliable braking performance is considered to be the most important target.

In real conditions, it is difficult to establish very accurate EMB models, and the cascaded PID control does not rely on accurate EMB models [24]. Mine electric vehicles usually need a larger friction braking force because of larger vehicle mass and load; meanwhile, the PID algorithm can not avoid increases in arising time and adjustment time as the demand of braking force increases. Therefore, a cascaded three close-loop PID controller with contact point detection technology is proposed at first, in which close-loop control of clamping force is optimized by a Variable Universe Fuzzy (VUF) algorithm with stretching factors.

In general, the main contributions of this paper are listed as follows:

- An EMB system model for target UETRV is established and analyzed.
- Based on established model, a novel VUF-PID clamping force controller with a cascade three-close-loop control architecture is designed, which can also achieve the function of fast and adaptive tracking of clamping force and autonomous adjustment of gap distance.
- A contact point detection method to determine control mode switch between gap distance control and clamping force control via an experimental method is proposed.
- Simulations and experiments in typical braking conditions are utilized to verify the above contents.

The rest of this paper is presented as follows. The next section introduces modeling and analysis of EMB. Section 3 presents the overall control system, involving the cascaded three-closed-loop PID control method with the contact point detection method utilized for force/position control mode switch. Especially, the loop of clamping force control utilizes a combination of the VUF algorithm and classic PID control, where a fuzzy controller adopts a stretching factor. The results and discussion of simulations in typical braking conditions are shown in Section 4. Correspondingly, the experimental results and discussion of the EMB prototype in real UETRVs are presented in Section 5. Finally, Section 6 describes the conclusions.

## 2. EMB Modeling and Analysis of UETRV

**Figure 1:**
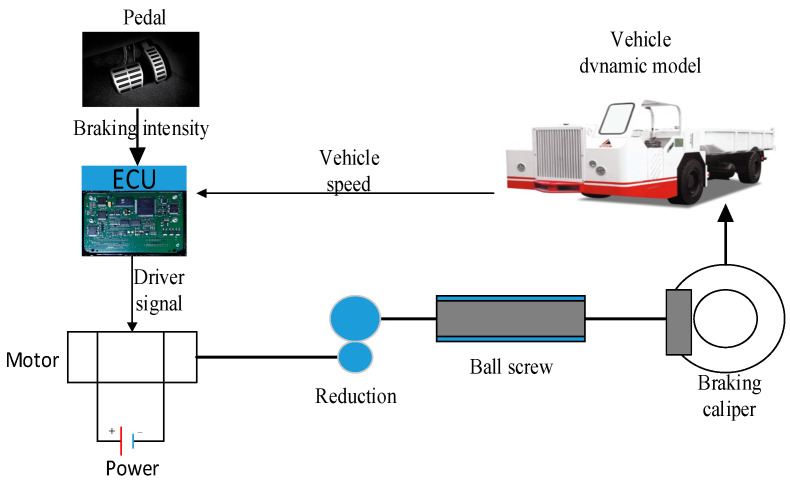
The schematic diagram of EMB for UTRVs.

A surface-mounted permanent magnet synchronous motor (SPMSM) is adopted to generate the initial force of the EMB because both a high power density and small size are needed by the EMB [25]. The other parts in this EMB include a planetary reduction gear, a ball screw, a pair of frictional pads and a braking disk. It is seen in Figure 1 that the driver steps down on the braking pedal to provide a braking intensity signal for the Electric Control Unit (ECU) of this EMB. The ECU calculates the braking force command according to the target braking intensity. Subsequently, the EMB controller receives a force command of every single EMB actuator and sends a Pulse Width Modulation (PWM) signal corresponding to braking force reference into the three-phase power inverter. And then the inverter provides a 3-phase sine wave input for the EMB motor to rotate. Further, the reduction gear and ball screw achieve the function of force enlarging and motion conversion, respectively. The head part in the ball screw directly compresses frictional pads onto the disk to make the vehicle decelerate and stop. In this work, the electro and mechanical subsystem model is discussed, respectively. 

### 2.1. Modeling of Motor

(1) $$\left[\begin{array}{c} f_{\alpha }\\ f_{\beta } \end{array}\right]=\left[\begin{array}{ccc} \begin{array}{c} 1\\ 0 \end{array} & \begin{array}{c} -1/2\\ \sqrt{3}/2 \end{array} & \begin{array}{c} -1/2\\ -\sqrt{3}/2 \end{array} \end{array}\right]\left[\begin{array}{c} f_{a}\\ f_{b}\\ f_{c} \end{array}\right]$$

(2) $$\left[\begin{array}{c} f_{d}\\ f_{q} \end{array}\right]=\left[\begin{array}{cc} \cos\theta_{e} & \sin\theta_{e}\\ -\sin\theta_{e} & \cos\theta_{e} \end{array}\right]\left[\begin{array}{c} f_{\alpha }\\ f_{\beta } \end{array}\right]$$

where $f_{a},f_{b},f_{c}$ are the vector components in three-phase ABC coordinates, respectively. $f_{\alpha },f_{\beta }$ are the vector components in two-phase α−β coordinates, respectively. $f_{d},f_{q}$ are the vector components in d−q rotary coordinates, respectively. $\theta_{e}$ is the electrical rotor angle. Therefore, the d−q voltage formula is rewritten as follows:

(3) $$\left\{\begin{array}{c} v_{d}=Ri_{d}+L_{d}\frac{di_{d}}{dt}-\omega_{e}\psi_{q}\\ v_{q}=Ri_{q}+L_{q}\frac{di_{q}}{dt}+\omega_{e}\psi_{d} \end{array}\right.$$

(4) $$\left\{\begin{array}{l} \psi_{d}=L_{d}i_{d}+\phi \\ \psi_{q}=L_{q}i_{q} \end{array}\right.$$

where $V_{d}$, $V_{q}$ is the d-axis and *q*-axis voltage; $i_{d}$, $i_{q}$ is the stator current in the d−q axis; $L_{d}$, $L_{q}$ are inductance in d−q coordinates; $\omega_{e}$ is the electro angle of motor $\Psi_{d};\;\mathrm{and}\Psi_{q}$ is the flux linkage in *d* − *q* coordinates and it can be expressed by $K_{e}=n_{p}\Psi_{d}$ where $K_{e}$ presents the back electromotive force constant and $n_{p}$ presents the pole pairs number. The relationship between the electrical angle speed $\omega_{e}$ and mechanical angular speed $\omega_{m}$ can be expressed as $\omega_{m}\times \omega_{e}=n_{p}\omega_{m}/2$; ϕ is the flux linkage of the rotor magnetic field; R presents the stator resistance. The SPMSM torque equation can be shown as Equation (5) [26].

(5) $$T_{m}=\frac{3}{2}n_{p}[\phi i_{q}+(L_{d}-L_{q})i_{d}i_{q}]$$

According to the SPMSM principle, if $i_{d}$ is fixed at zero, the torque will go up to the maximum value [27]. Hence, Equation (5) can further become a simple form similar to the DC motor torque formula in which currents can have a linear expression with torque:

(6) $$T_{m}=\frac{3}{2}n_{p}\phi i_{q}$$

where a torque constant $K_{t}$ further replaces $\frac{3}{2}n_{p}\phi$. Therefore, Equation (6) can again be simply expressed as follows:

(7) $$T_{m}=K_{t}i$$

### 2.2. Model of Mechanical Components

#### 2.2.1. Mechanical Transmission Model

The reduction gear is utilized to enlarge the force generated from the motor. Subsequently, the ball screw can generate linear motion of the ball screw nut which directly generates clamping force on the pad. The position of the screw head can be controlled by adjusting the motor rotor angle displacement because there is a relatively linear mapping relationship between them. The load torque $T_{L}$ is generated from the clamping force between the pads and the disk and can be calculated as follows:

(8) $$T_{L}=\frac{1}{2\pi }\frac{L_{s}}{\eta_{g}n_{g}}\frac{1}{\eta_{s}}F_{cl}$$

where $\eta_{g}$ presents the efficiency coefficient of the gearbox and $\eta_{s}$ presents the one of the ball screw; $n_{g}$ is the reduction ratio of the gear; and $L_{s}$ is the lead of the ball screw. $F_{cl}$ is the clamping force between the braking pad and disc, which is the key controlled object. Equation (8) is usually expressed as $T_{L}=k_{cl}F_{cl}$, in which $k_{cl}$ is the total gearing transfer gain. 

The mechanical torque of braking motor $T_{m}$ should be specifically expressed as Equation (9) for further controller design:

(9) $$T_{m}=J_{m}\frac{d\omega_{m}}{dt}+B_{f}\omega_{m}+T_{L}$$

where $\omega_{m}$ is the mechanical angle velocity; $J_{m}$ is the rotor inertia constant; $B_{f}$ is the damping coefficient of the rotor.

#### 2.2.2. Load Model

The total resistance exerted by the actuator drive mechanism on the motor output shaft is motor load $T_{L}$. $F_{cl}$ can be obtained by a fitting equation [28] as Equation (10):

(10) $$F_{cl}=A_{1}s^{3}+A_{2}s^{2}+A_{3}s$$

where $A_{1}$, $A_{2}$, and $A_{3}$ are the fitting coefficients; s is the deformation of the braking disk and also the displacement of screw heads, mm.

#### 2.2.3. Brake Disc Model

The brake disc is a clamping mechanism for generating clamping force. Under the clamping action of the brake disc, the left and right sides of the brake disc produce the same friction torque. The friction torque $T_{\mu }$ of the brake disc of the single-wheel EMB is as follows:

(11) $$T_{\mu }=2F_{cl}\cdot R_{b}\cdot \mu_{b}$$

where $F_{cl}$ is the clamping force, N; $\mu_{b}$ is the friction coefficient of the friction plate; $R_{b}$ is the effective radius of the brake disc, m.

**Figure 2:**
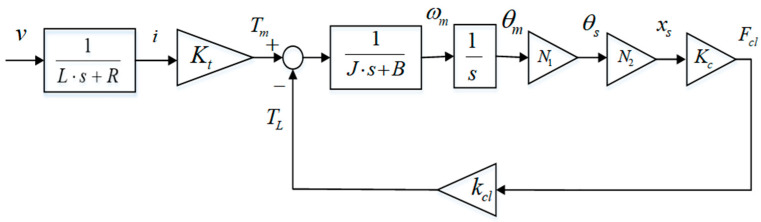
EMB actuator basic model.

Based on the above contents, a mathematical model of an EMB actuator for a target UETRV is described in Figure 2. The relationship between the current and voltage is simplified as a one-order transfer function with parameter L and R. The ball screw angle $\theta_{s}$ and its displacement $x_{s}$ have a proportional relationship expressed as $x_{s}=N_{2}\theta_{s}$. $N_{2}=L/2$, where L is the lead of the ball screw. In this model, mapping the relationship curve between $x_{s}$ and $F_{cl}$ is considered as a cubic polynomial, as shown in Equation (10).

**Figure 3:**
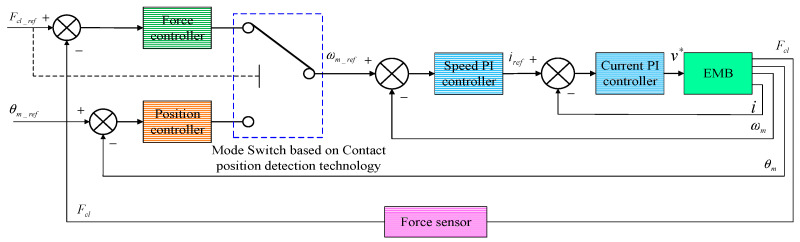
Architecture of cascaded EMB control system.

This EMB model is adopted as a basic model of EMB actuator when tracking clamping force, which is also packaged as a subsystem of the overall EMB control system in Figure 3.

## 3. Design of Controllers

In this section, a cascaded control architecture with three close-loop PID control units is described in detail. Especially, the clamping force is tracked reliably via adopting a Variable Universe Fuzzy (VUF)–PID algorithm, which can adaptively adjust gain parameters of $∆k_{p}$, $∆k_{i}$, $∆k_{d}$ of the clamping force loop and reduce adjustment time compared with only the PID control. The contact point detection technology based on the experimental method is also illustrated in this section.

### 3.1. Cascaded Three Close-Loop Force/Position Control Architecture

The overall cascaded PID control architecture is shown in Figure 3. 

It can be seen in Figure 3 that there are four sub-controllers in this overall EMB system. Two PI feedback controllers with different gain parameters are utilized for the current control loop and speed control loop of this EMB system, respectively. Considering the important effect of the clamping force variation, both the clamping force controller and the gap distance controller adopt proportional-integral-differential (PID) control, which is used to provide the target angle velocity of motor for the subsequent speed control loop. The reference command $F_{cl\_ref}$ and $\theta_{m\_ref}$ are constant numbers which are independent of time, because when the vehicle is in process of braking, a fixed braking clamping force must act on the friction pad of the brake after a constant motor angle displacement corresponding to the braking gap distance, which can be tracked in a timely manner so as to eliminate the braking gap. The position controller is only adopted to adjust the expected braking gap distance by utilizing the relationship between the motor angular position $\theta_{m}$ and the stoke displacement of screw nut $x_{s}$ as shown in Equation (12). With this contact point detection method, this proposed control system of the EMB system can automatically switch operation stages between gap elimination, tracking clamping force and gap recovery.

After the local EMB controller detects the non-zero measured value from the force sensor, the force controller, in a timely manner, take over the permissions of system control to work.

(12) $$x_{s}=\theta_{m}\cdot n_{g}\cdot L_{s}/2\pi$$

The gain parameters of current, speed and force loops are tuned in the Simulation platform in the first stage and then optimized on an experimental prototype in the second stage based on the experienced tuning method. After that, the achieved parameters can be set as proper gain parameters for the current loop and speed loop of motor and the position control loop. And the achieved parameters for the clamping force control loop just become the initial value of $K_{P0}$, $K_{i0}$, $K_{d0}$ of the VUF-PID controller for controlling the clamping force.

#### 3.1.1. Current Loop

**Figure 4:**
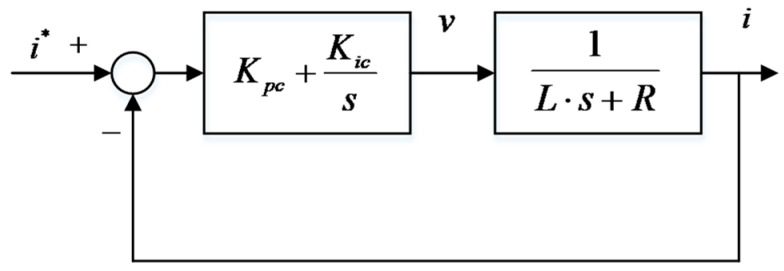
Current control loop.

The current close-loop PI controller is shown in Figure 4. Equation (13) is its open-loop transfer function.

(13) $$G_{c}^{o}(s)=\frac{K_{pc}s+K_{ic}}{s}\frac{1}{Ls+R}=\frac{K_{pc}\left(s+\frac{K_{ic}}{K_{pc}}\right)}{Ls\left(s+\frac{R}{L}\right)}$$

Equation (14) is the expression of the current PI controller.

(14) $$\frac{I(s)}{I^{*}(s)}=G_{c}^{c}(S)=\frac{\frac{K_{pc}}{L}}{s+\frac{K_{pc}}{L}}$$

#### 3.1.2. Speed Loop

**Figure 5:**
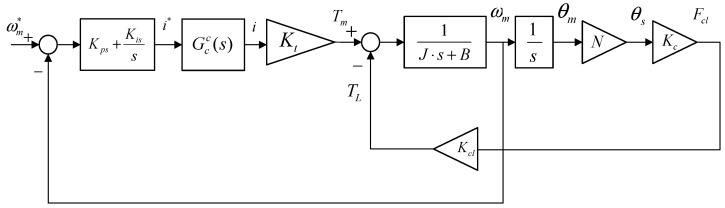
Speed control loop with a detailed EMB model.

The speed PI controller with a detailed EMB model is shown in Figure 5.

#### 3.1.3. Basic Force PID Controller

**Figure 6:**
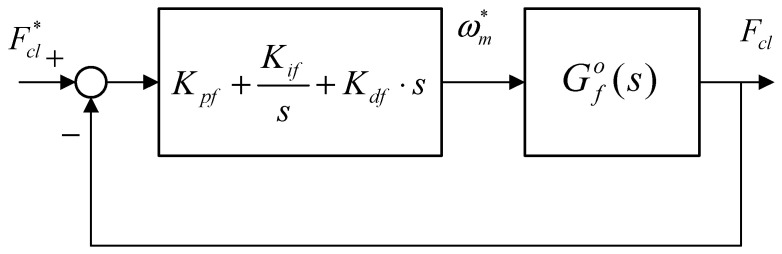
Clamping force close-loop control system.

The clamping force control of this EMB actuator which outputs the reference speed is based on a VUF-PID controller. The diagram of clamping force close-loop PID is shown in Figure 6, where $G_{f}^{o}(s)$ is described as Equation (16).

(15) $$G_{f}^{o}(s)=\frac{K_{ps}\cdot K_{t}\cdot K_{pc}\cdot N\cdot K_{c}/L\cdot s+K_{is}\cdot K_{t}\cdot K_{pc}\cdot N\cdot K_{c}/L}{J\cdot s^{4}+(B+K_{pc}\cdot J/L)\cdot s^{3}+(\cos\alpha \cdot r\cdot K_{c}\cdot N^{2}+K_{pc}\cdot B/L+(K_{ps}\cdot K_{t}\cdot K_{pc}/L))\cdot s^{2}}\;+((K_{pc}/L)\cdot \cos\alpha \cdot r\cdot K_{c}\cdot N^{2}+(K_{is}\cdot K_{t}\cdot K_{pc}/L))\cdot s$$

where α is the ball screw thread angle, and r is the thread length.

### 3.2. Variable Universe Fuzzy–PID Controller for Force Control Loop

In order to obtain a faster response performance, an VUF algorithm is designed for the force control loop of the designed EMB, which makes the designed EMB to rapidly and stably track the target clamping force.

In the time domain, the target clamping force command of the controlled system is y(t), the actual response value is r(t), and the deviation between them is error e(t), e(t)=y(t)−r(t). The output of classic PID controller is calculated based on $e\left(t\right),$ as follows:

(16) $$u(t)=K_{p}e(t)+K_{i}\int_{0}^{t}e(t)dt+K_{d}\frac{de(t)}{dt}$$

in which $K_{p0}$, $K_{i0}$, and $K_{d0}$ are three gain parameters, respectively.

Utilizing the fuzzy algorithm to calculate the parameter adjustment quantities of the PID controller in the clamping force control loop, the parameters of the fuzzy controller and PID controller can be adjusted in a timely manner based on the feedback quantities of error e and error change rate ec. The domains of e and ec are [−24,000, 24,000] and [−2400, 2400], and the domains of ∆*K_p_*, ∆*K_i_*, ∆*K_d_* are [−1, 1], [−0.1, 0.1] and [−0.002, 0.002]. The PID parameter correction values $∆K_{p}$, $∆K_{i}$, $∆K_{d}$ are the output of the controller based on Mamdani fuzzy reasoning rules.

Transfer of the two-dimensional value from the precise field to the fuzzy field is achieved. The coefficient factors of e, ec are $K_{e}$, $K_{ec}$, and the scaling factors of correction quantities $∆K_{p}$, $∆K_{i}$, $∆K_{d}$ are $K_{up}$, $K_{ui}$, $K_{ud}$. The PID parameter adjustment quantities $∆K_{p}$, $∆K_{i}$, $∆K_{d}$ can be calculated from the fuzzy calculation unit. The fuzzy rules tables of controller parameters are given in Table 1, Table 2 and Table 3, which are first determined by expert experience and knowledge and tuning parameters via simulation. The seven fuzzy language variables are as follows: NB is Negative Big, NM is Negative Medium, NS is Negative Small, ZE is Zero, PS is Positive Small, PM is Positive Medium, and PB is Positive Big, which are the key values of the fuzzy calculation unit.

And the updated gain parameters of the PID controller are determined by Equation (17).

(17) $$\left\{\begin{array}{l} K_{p}=K_{p0}+\Delta K_{p}K_{up}\\ K_{i}=K_{i0}+\Delta K_{i}K_{ui}\\ K_{d}=K_{d0}+\Delta K_{d}K_{ud} \end{array}\right.$$

in which $K_{p0,\;}K_{i0},\;K_{d0}$ are obtained from initial gain parameters or the last sample time step.

To avoid the phenomenon of prolonging the response time caused by redundant rules of the normal Fuzz-y–PID algorithm, based on basic fuzzy–PID control, a scaling factor is added to adjust the system rapidly and shorten the adjustment time when applied for large braking force demand. 

**Figure 7:**
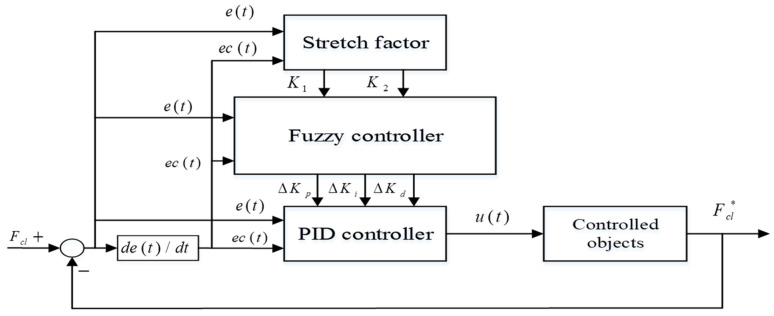
Proposed clamping force PID controller based on VUF algorithm.

In this paper, the designed VUF-PID algorithm is just utilized to adjust $K_{p}$, $K_{i}$. The VUF-PID algorithm in Figure 3 is presented in Figure 7. $K_{1},K_{2}$ are the linear transfer factors acting on the output of the proposed Variable Universe Fuzzy algorithm. 

There are two usual methods for designing scaling factors: one is the adaptive function method and the other is fuzzy language. The adaptive function method has advantages of a simple structure and easy control, which is not suitable for complex and practical engineering problems. As a result, the fuzzy language is selected to scale the domain.

As shown in Figure 7, the clamping force error e(t) and its changing rate ec(t) are input signals of both the fuzzy controller and stretch factor calculator. The output signals of the proposed VUF algorithm are $∆K_{p},∆K_{i},∆K_{d}$ and these can be adopted to adjust the three gain parameters of the basic PID controller. The target current signal u(t) directly acting on the controlled objects (namely EMB actuator) is the output of the proposed VUF-PID controller; meanwhile, the output of the “controlled objects” is target clamping force $F_{cl}$. This total controller system can adaptively adjust the controller parameters to track the target clamping force $F_{cl}^{*}$.

### 3.3. Estimation Method of Contact Point Utilized for Force/Position Switch

It is key to estimate the critical contact point (CCP) of the pad and disk for the transient switch process between the force control mode and position control mode. The motor of EMB always rotates relatively fixed revolutions after the pads really begin to make contact with the braking disk. After this contact point, there is also a mapping relationship between the motor revolutions and the clamping force. Therefore, it is able to detect CCP by tracking motor revolutions. The relationship curve between the positive compressing force and motor rotation angle position is considered as the below form.

(18) $$F_{cl}=g(\theta_{m})$$

**Figure 8:**
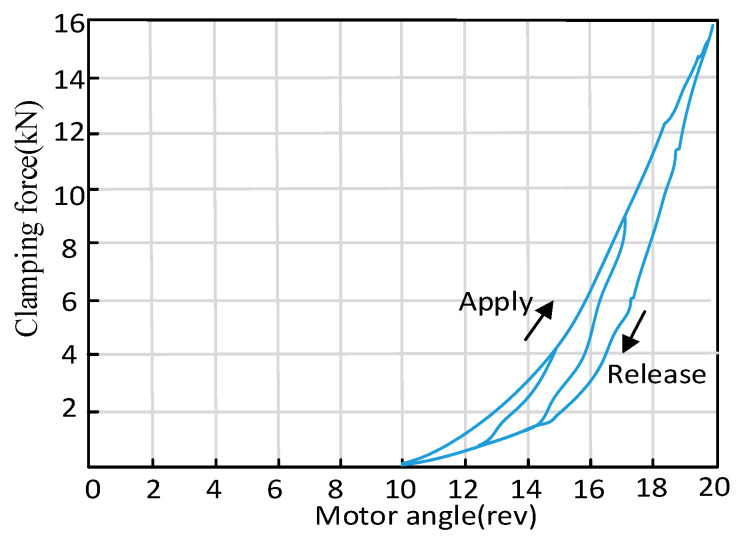
Clamping force test for contact point detection.

Equation (18) is a curve with multiple segments and high nonlinear characters, as shown in Figure 8.

In this paper, motor revolutions corresponding to the CCP are achieved via an experimental method. Aimed at obtaining the motor revolutions’ value, which presents the initial contact position of this EMB, a force sensor is used to provide a feedback signal to detect the relationship between the motor rotary motion and actual clamping force. The test value of clamping in this test is set as 16 kN. The motor revolutions range from 0 to 20. The measured signals include clamping force signal from the force sensor ranging from 0 to 30 kN, motor revolutions from the encoder with a precision of 360 count per turn and the motor current signal from the current sensor ranging from 0 to 30 A. The critical revolutions are derived from this method when the contact is initially 10, as shown in Figure 8. The corresponding switch time is only 0.95 s, which is also mentioned in the subsequent experiments section.

## 4. Simulation Results and Discussion

**Figure 9:**
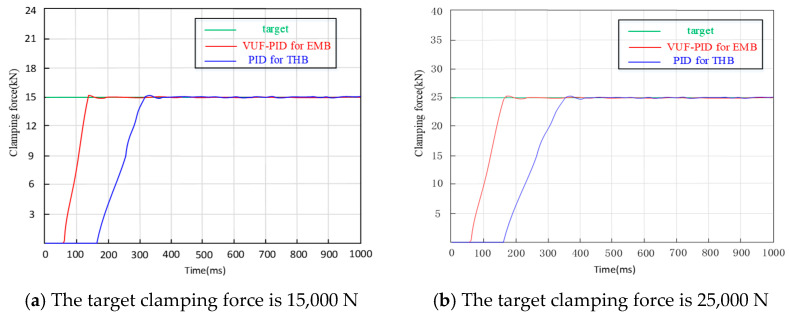
Simulation of step signals.

**Figure 10:**
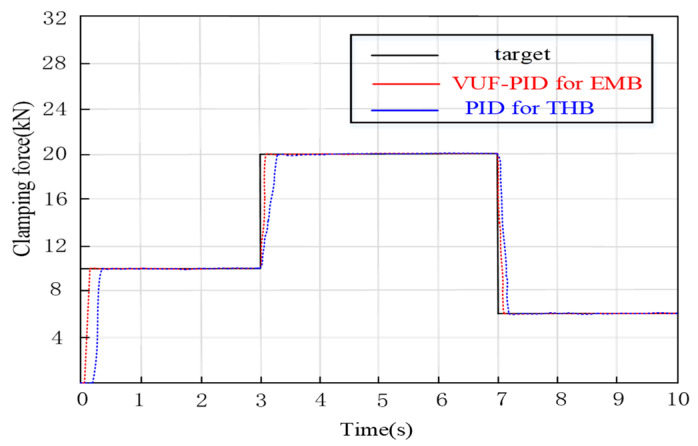
Gear switching braking condition.

**Figure 11:**
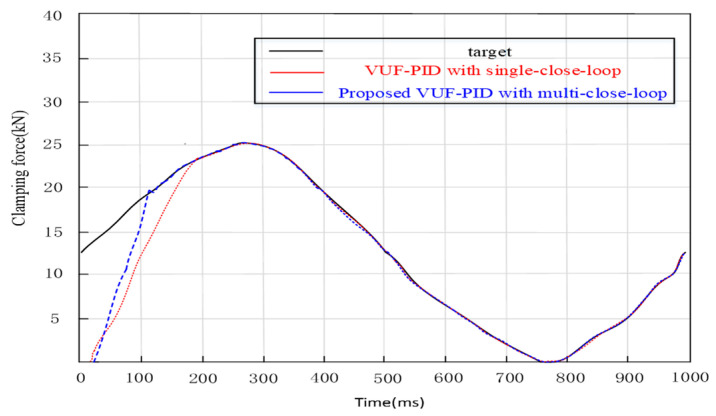
Sine braking condition.

The VUF-PID parameters are listed in Table 4. The values in Table 4 are derived from experience tuning parameters and further corrected in this version in manuscript by the tuning parameters in the process of the simulations. Table 5 shows the parameters of the EMB model. The comparison between VUF-PID and PID is analyzed by simulations in Matlab/simulink v.2020. The results with three kinds of clamping force input signals are presented in Figure 9, Figure 10 and Figure 11.

(1) Simulation results and discussion of step braking condition response

The inputs of 15 kN and 25 kN are simulated in Figure 10 with an initial gap of 0 mm. In comparison of Figure 9a,b, the simulation evaluation parameters of the two EMB control strategies and the THB control strategy are shown in Table 6.

In Table 6, the adjustment time of VUF-PID with multiple close-loops is 0.02 s faster than VUF-PID control with a single close-loop. And the overshoot of the proposed multi-close-loop control with optimization of the VUF algorithm also decreases 10% compared with the single-close-loop VUF-PID controller.

It is obviously seen that the response speed of the proposed EMB control strategy is 0.18 s faster than THB, at least when inputting these two-step braking commands. The three VUF-PID controllers designed for the EMB actuator in this paper can shorten the response time and adjustment time of the EMB system for UETRV and enhance the stable performance of the clamping force tracking, which can provide a practical useful research basis for accurate motion control, including a precise stopping distance and smooth deceleration performance. According to a coal mine transportation safety criterion of China, considering a normal driving speed at value of 7 m/s, this VUF-PID-based EMB control strategy can shorten the stopping distance by 1.26 m at least, which is also useful for precising parking and emergent obstacle avoidance of UETRVs.

(2) Gear switching braking condition simulation and discussion

Braking force may always switch between different braking intensities by the driver or, in autonomous driving, the decision system, which is called gear switching braking. The target input step values are 0−10 kN−20 kN−6 kN. Figure 10 illustrates the clamping force output of gear switching braking.

In Figure 10, the establishment times of a multi-close-loop VUF-PID controller and single-close-loop VUF-PID are 0.145 s (steady-state error: 0.21%), and 0.168 s (error: 0.28%), respectively. When the clamping force is increased from 10,000 N to 20,000 N, the clamping force adjustment times of the multi-close-loop VUF-PID and single-close-loop VUF-PID are 0.081 s (steady-state error: 0.18%), and 0.102 s (steady-state error: 0.24%). In the process of actual clamping force decreasing from 20,000 N to 6000 N, the adjustment times of multi-close-loop VUF-PID and single-close-loop VUF-PID are 0.066 s and 0.088 s, respectively. Both of them have smaller adjustment times compared with the stage of clamping force jumping. It also can be seen that the VUF-PID controller presents not only the smaller dead-zone time but also smaller adjustment time compared with the single-close-loop VUF-PID.

(3) Sine braking condition simulation and discussion

The Sine input is the expression u(t)=|25000sine(2πt)(N)|. The response is depicted in Figure 11. The adjustment time of the proposed multi-close-loop VUF-PID is 0.127 s and the adjustment time of the single-close-loop VUF-PID is 0.168 s. It is obviously seen that the proposed EMB system is 0.04 s faster than the single-close-loop VUF-PID strategy because of the intrinsic advantages of pure electro-mechanical transmission. These results indicate that the multi-close-loop VUF-PID can effectively track the Sine braking signal. Reducing the response time of the EMB is very important to enhance the vehicle‘s stability performance from the anti-lock braking system (ABS) when the UETRV drives in the wet and slippery tunnel road.

(4) Comparison between proposed VUF-PID controller and Fuzzy–PID controller

**Figure 12:**
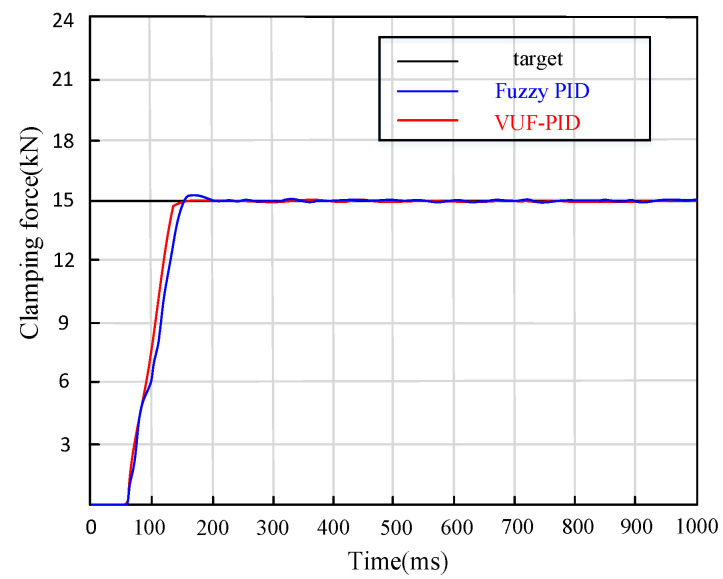
Comparison between proposed VUF-PID and Fuzzy–PID under stepping braking.

Step signals of 15,000 N are applied to the input end of the target clamping force of the EMB actuator. The simulation curves of clamping force response in the control system are shown in Figure 12.

It can be seen in Figure 12 that the adjustment time of Fuzzy–PID control is 0.223 s and the overshoot is 1.52%. The maximum adjustment time of the proposed multi-close-loop VUF-PID is 0.16 s and the overshoot is 0.15%. The proposed VUF-PID controller designed in this paper not only avoids the oscillation problems of basic PID control but also responds faster than the Fuzzy–PID controller by 0.6 s and reduces the overshoot by more than 80%. In general, the adjustment time and overshoot of the EMB system are effectively reduced, and the dynamic- and steady-state performance are significantly improved.

## 5. Experimental Results and Discussion

### 5.1. Experimental Platform

**Figure 13:**
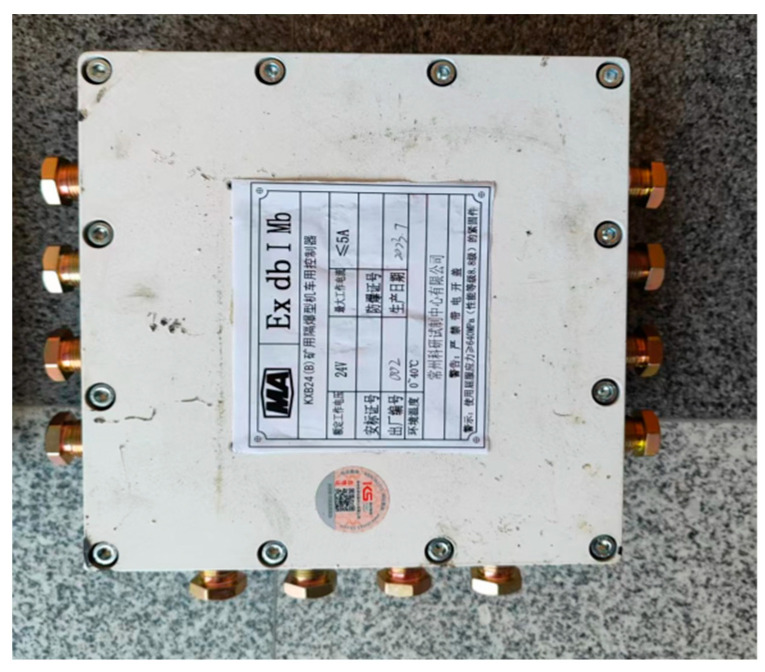
Developed ECU of EMB with explosion-proof processing.

In this work, it is seen in Figure 13 that an experimental EMB prototype controlled by an Electrical Control Unit (ECU) with explosion-proof processing is established according to safety standards of explosive atmospheres in underground coal mines. Figure 13 shows the explosion-proof EMB controller. The metal terminals are the only electric signal transmission interface of the inner electric parts in this EMB controller. The core of this EMB controller is an Infineon high-performance micro-processor (32 bits) with many integrated interface circuits and meets the highest level D of commercial vehicle safety standards. 

**Figure 14:**
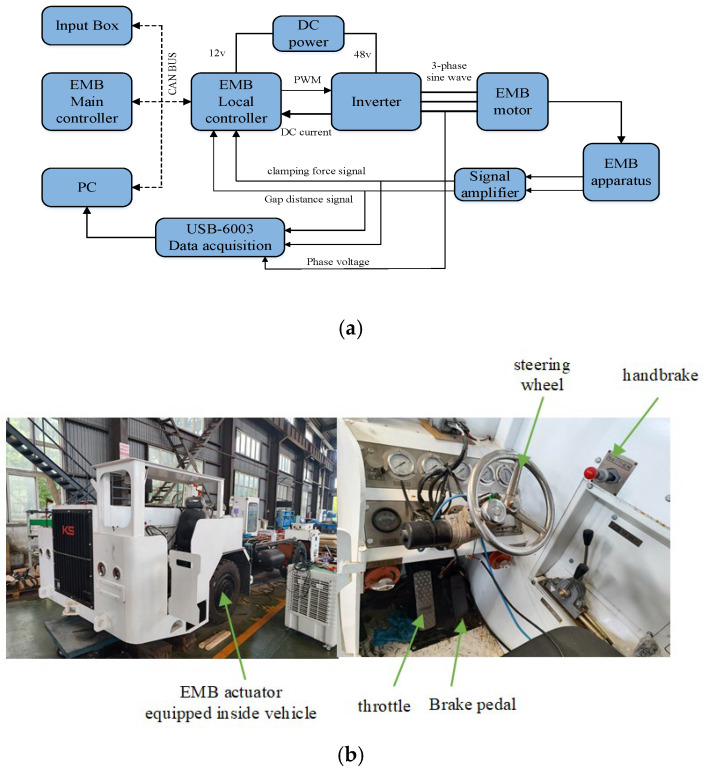
Experimental setup. (**a**) Proposed EMB system block diagram; (**b**) test vehicle equipped with designed EMB.

The system architecture of this prototype for testing in real UETRVs is presented in Figure 14a. A surfaced PMSM (400 W (3.5 N·m) supplied by a three-phase inverter power with a DC input of 48 V voltage is adopted as the initial braking force source of the EMB system. The measurement sensor of the motor current ranges from 0 to 30 A. The clamping force sensor ranges from 0 to 30 kN. All of these signals can simultaneously feedback into the EMB local controller to achieve close-loop tracking control and could also be collected in PC monitor software via a high-speed data collector. The master controller of the EMB managing the whole distributed braking system sends the target braking force command to the local controller (only one of four is shown in Figure 14a as a example). The overall communication mode between every part of this prototype is Controller Area Network (CAN). The computer can also monitor and control the detailed operation process of this prototype. The real vehicle test is conducted and implemented in test field of Keshi Group in Changzhou, China, as shown in Figure 14b.

The components selection of experimental prototype is also based on the parameters in Table 5.

### 5.2. Experimental Results and Discussion

#### 5.2.1. Brake Clearance Recovery Test

The experimental initial state is that the distance between friction pad and disk is zero mm. The target clearance is set to 1 ± 0.02 mm. When the test is started, the braking command value is sent into the local controller. The EMB with three-close-loop PID control can track the input reference clearance value within 95 ms and be kept stable.

**Figure 15:**
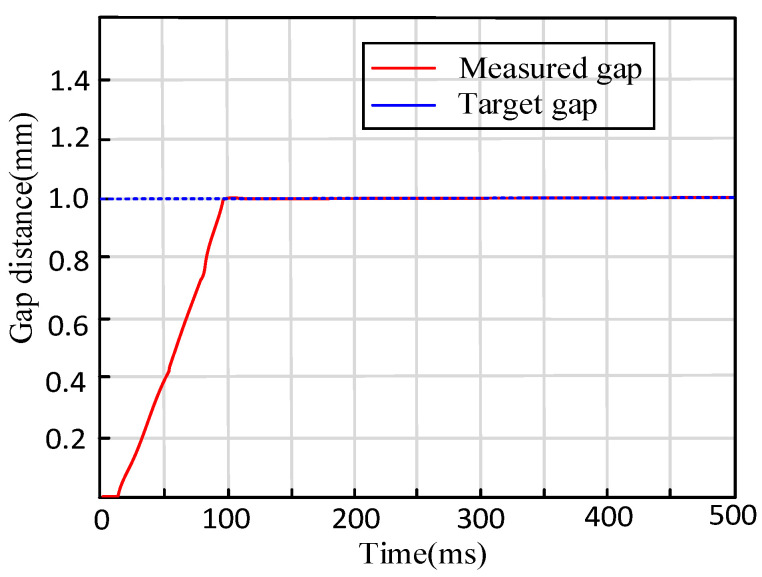
Brake clearance recovery experimental curve.

It is shown in Figure 15 above that the initial 30 ms is the dead zone of the EMB and the data collector does not obtain any effective sensor profiles. After this dead zone, the braking motor begins to rotate. The overall adjustment time of braking clearance is 95 ms. After 0.1 s, the stead error range is less than 0.02 mm. These performance indexes can also meet the braking safety criterion in coal mines.

#### 5.2.2. Brake Clamping Force Test

(1) Step braking condition experiments

**Figure 16:**
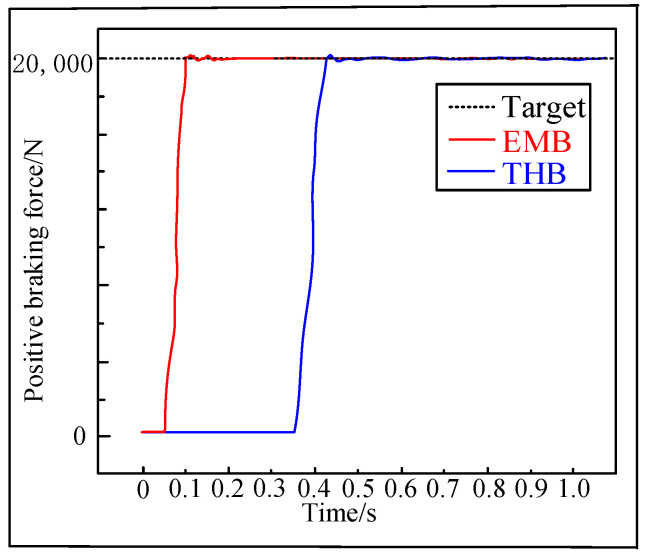
Clamping force–step response curves comparison between EMB with VUF-PID and THB with classic single-close-loop PID.

It is a normal test to compare the braking clamping force–step response curves between the EMB and THB in Figure 16. According to the whole vehicle parameters of light UETRVs, the maximum value of brake clamping force $F_{nmax}$ is calculated as 60,000 N. Considering that the normal braking intensity of this target UETRV is approximately 1/3, it is normal to set the typical step response input value of clamping force at 20,000 N.

The force response curve of the VUF-PID controller for the proposed EMB displays that after the dead zone time (0.035 s), the clamping force curves begin to raise. The system adjustment time is 0.208 s, which is sufficient to meet braking safety requirements of low-speed mine vehicles, and the overshoot is 0.21%. After an adjustment time, the steady-state mean value of the EMB reaches a value of 19,783 N, and the error is 217 N (1.1%). The adjustment time of THB is 0.37 s and the steady-state mean value of THB reaches a value of 19,958 N, and the error is 282 N (1.33%). It is obviously seen that the proposed EMB system has a smaller braking acting time and achieves a shorter braking distance compared with the THB applied in UETRVs currently when dealing with the same braking working conditions, which means better vehicle safety and precise parking of automated UETRVs.

(2) Gear switching braking condition

When the UETRV brakes, it is a actual situation that the driver will change the braking intensity level. Hence, the EMB system still needs to rapidly track the required input. The experimental conditions are as follows: applying brake gear 0–10,000–20,000 N–6000 N.

**Figure 17:**
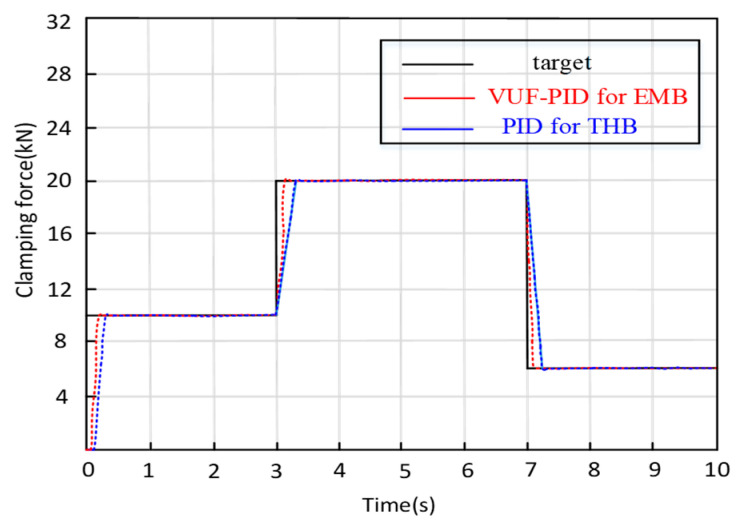
Gear switch braking condition.

Figure 17 displays the test curves of clamping force under switching braking conditions. When the input changes from 0 to 10,000 N, the adjustment times of the multi-close-loop VUF-PID and single-close-loop VUF-PID of EMB are 0.132 s and 0.167 s. When the clamping force ranges from 10,000 N to 20,000 N, the clamping force response times of both the multi-close-loop VUF-PID and single-close-loop VUF-PID are 0.071 s and 0.092 s, respectively. It can be seen that the proposed multi-close-loop VUF-PID can track the updated target input rapidly and stably under braking switching conditions, especially in the transition stage of switching braking command. In the transition process, the EMB controlled by the multi-close-loop VUF-PID has a smaller adjustment time, which benefits from the adaptive adjustment ability of parameters $∆K_{p}$, $∆K_{i}$ and $∆K_{d}$ of the VUF-PID and the comprehensive control of the multi-close-loop in conditions of unmodeled dynamics compared with the single-close-loop VUF-PID algorithm. Compared with the response time 0.37 s of THB in current engineering applications, this proposed controller shows obvious advantages in terms of clamping force response performance.

## 6. Conclusions

In this paper, an EMB composed of a motor, a reducer, a ball screw, a pad and a disc is designed, which eliminates all the hydraulic components of the THB system. The single-closed-loop VUF-PID controller is extended to multi-closed-loop control, and the EMB is controlled by the effective force/position switching method. The fuzzy field can be adjusted in real time to improve the adaptability of the system. Finally, the tracking effects in three conditions are proved. According to the experimental results and regulations of coal mine safety transportation, this EMB system has a response time less than 0.13 s and gap elimination time of 0.09 s, and it can meet braking safety requirements. The enhanced control precision of the clamping force can benefit precise and stable motion control of automated UETRVs or other lower-speed engineering vehicles.

In future research work, it is necessary to consider an estimation method of clamping force in case of potential failure of clamping force sensors because of seriously wet and dusty environments in underground coal mine environments, which can obviously enhance the reliability of the EMB system in real engineering application. In addition, it is also a valuable idea to improve the performance of deceleration stability by adopting this clamping force controller as the lower clamping force controller so that the constant deceleration controller can achieve more precise and stable deceleration while reducing the oscillation of the braking clamping force.

## Figures and Tables

**Table 1:** The fuzzy rules control table of $∆K_{p}$.

e	ec						
	NB	NM	NS	ZE	PS	PM	PB
NB	NB	NB	NM	NM	NS	ZE	ZE
NM	NB	NB	NM	NS	NS	ZE	ZE
NS	NB	NM	NS	NS	ZE	PS	PS
ZE	NM	NM	NS	ZE	PS	PM	PM
PS	NM	NS	ZE	PS	PS	PM	PB
PM	ZE	ZE	PS	PS	PM	PB	PB
PB	ZE	ZE	PS	PM	PM	PB	PB

**Table 2:** The fuzzy rules control table of $∆K_{i}$.

e	ec						
	NB	NM	NS	ZE	PS	PM	PB
NB	PB	PB	PM	PM	PS	ZE	ZE
NM	PB	PB	PM	PS	PS	ZE	NS
NS	PM	PM	PM	PS	ZE	NS	NS
ZE	PM	PM	PS	ZE	NS	NM	NM
PS	PS	PS	ZE	NS	NS	NM	NM
PM	PS	ZE	NS	NM	NM	NM	NB
PB	ZE	ZE	NM	NM	NM	NB	NB

**Table 3:** The fuzzy rules control table of $∆K_{d}$.

e	ec						
	NB	NM	NS	ZE	PS	PM	PB
NB	PS	NS	NB	NB	NB	NM	PS
NM	PS	NS	NB	NM	NM	NS	ZE
NS	ZE	NS	NM	NM	NS	NS	ZE
ZE	ZE	NS	NS	NS	NS	NS	ZE
PS	ZE	ZE	ZE	ZE	ZE	ZE	ZE
PM	PB	NS	PS	PS	PS	PS	PB
PB	PB	PM	PM	PM	PS	PS	PB

**Table 4:** Parameters of the VUF-PID controller.

Parameter	Symbol	Value	Parameter	Symbol	Value
Initial value of the scale factor	$K_{p0}$	9.5	Quantification factor of ∆e	$K_{ec}$	0.0035
Initial value of the integral factor	$K_{i0}$	0.03	Scale factor of $∆K_{p}$	$K_{up}$	0.18
Initial value of the differential factor	$K_{d0}$	0.003	Scale factor of $∆K_{i}$	$K_{ui}$	0.018
Quantification factor of e	$K_{e}$	0.00035	Scale factor of $∆K_{d}$	$K_{ud}$	0.00036

**Table 5:** Specific parameters of the EMB model.

Symbols	Physical Meanings	Values	Unit
R	Motor resistance	10.2	Ω
$L_{d},L_{q}$	Motor inductance	0.075	H
$J_{m}$	Rotor inertia	33.8	$g\cdot {cm}^{2}$
$B_{m}$	Motor damping	0.21	Ns/m
$K_{e}$	Back-emf constant	0.054 V·s/rad	V·s/rad
$K_{m}$	Motor torque constant	0.509	Nm/A
$n_{p}$	Number of pole pairs	2	-
$P_{s}$	Pitch of ball screw	5	mm
$e_{s}$	Efficiency of ball screw	92%	-
$n_{g}$	Total gear ratio	86:1	-
$e_{g}$	Efficiency of reduction gear	90%	-
$i_{v}$	Planetary gear reduction ratio	16	-

**Table 6:** Simulation results for two types of braking control strategies.

Target Clamping Force(N)	VUF-PID with Single-Close-Loop		VUF-PID with Multi-Close-Loop	
	Adjustment Time	Overshoot	Adjustment Time	Overshoot
15,000	0.183 s	0.40%	0.162 s	0.36%
25,000	0.220 s	0.35%	0.198 s	0.31%

## Data Availability

The data that support the findings of this study are available from the corresponding author upon reasonable request.
